# PP49 Differences And Inequalities In Drug Coverage Between The Public And Private Sectors Of The Brazilian Health System

**DOI:** 10.1017/S0266462325102687

**Published:** 2025-12-29

**Authors:** Elene Paltrinieri Nardi, Adriane Lopes Medeiros Simone, Ricardo Rodrigues Teixeira, Daniela Melo

## Abstract

**Introduction:**

Although Brazil has a public and universal health system, the Unified Health System (SUS), more than 50 million people also have private health insurance. This can generate health inequalities between citizens with and without private health insurance. We aimed to identify potential health inequalities by mapping the differences between drugs covered in the SUS and in the private health sector in Brazil.

**Methods:**

We compiled a list of new drugs, including biologics and advanced therapy products approved in Brazil (2010 to 2020) and analyzed drug-indication pairs from their labels. Oncology drugs were excluded. Coverage in the SUS and private health system was assessed through official documents (until 2022). Potential health inequalities were identified when pairs available to private health had no therapeutic alternatives in SUS care pathways.

**Results:**

A total of 206 drug-indication pairs were evaluated: 126 (61%) were not incorporated into either the SUS or private health insurance; 38 (18%) were exclusive to the SUS; 18 (9%) were exclusive to private health insurance; and 24 (12%) were in both. The SUS provided treatments for chronic non-communicable diseases, immune-mediated inflammatory diseases (IMIDs), rare diseases, and infectious diseases, while private health insurance primarily focused on IMIDs, particularly severe forms. Potential health inequalities were identified in four of the 18 pairs covered only by private health insurance, which were related to macular edema, asthma, thromboembolic disease, and anemia. Despite this, the SUS offered broader care pathways than private health insurance in most cases.

**Conclusions:**

Although some potential health inequalities were identified, the SUS incorporated more drugs for a wider variety of diseases than did private health insurance. Guided by the basic principles of universality, integrality, and equity, the SUS offers more comprehensive care than private health insurance and, in most cases, addresses gaps in care left by the private sector.

